# Lego Serious Play: Building engagement with cell biology

**DOI:** 10.1002/bmb.21608

**Published:** 2022-01-31

**Authors:** Claire Louise Palmer Garden

**Affiliations:** ^1^ School of Applied Sciences Edinburgh Napier University Edinburgh Scotland

**Keywords:** active learning, cellular biology, learning techniques methods and approaches, teaching

## Abstract

A Lego Serious Play (LSP) ‐ based exercise was developed to support student engagement with learning consolidation at the end of a first‐year undergraduate cell biology course. The exercise was offered in addition to a regular revision session in preparation for the summative exam. Students were studying four‐year BSc (Hons) degrees in: Animal Biology, Environmental Biology, Marine and Freshwater Biology, Biological Sciences, Biomedical Sciences, Microbiology & Biotechnology in Scotland, UK. Although many students studied Human Biology at High School, in‐depth cell biology was studied for the first time by the majority of students during this course. The LSP process was adapted for use in the classroom. Core concepts were identified from the twelve‐week cell biology course as the basis for LSP build challenges and incorporated into LSP build – share – reflect cycles by students individually and then joined together by the group to explore the interconnected nature of cell biology processes. Student and lecturer evaluations were thematically analyzed to explore the impact of the technique on student engagement. Results indicate that the method supports student cognitive and affective engagement who report improved and understanding of the topic, and enjoyment and interest. In addition, behavioral engagement such as learner interaction, independence, and empowerment were revealed by the lecturer interview. Identified barriers to the adoption of LSP include perceived issues around creativity, play and exploration and scientific identity, together with a lack of evidence of efficacy. This study seeks to remedy that gap.

## 1 INTRODUCTION

Evidence that Lego has been used as a teaching tool in Science, Technology, Engineering and Mathematics (STEM) subjects at advanced levels reaches back nearly two decades.^1^, ^2^ During this time its use has largely been restricted to robotic simulations to teach fundamental STEM concepts and computer programming, and literal model building (e.g.,^3^, ^4^). The strength of Lego models for teaching dynamic concepts lie in their modifiability, for example for teaching evolution and development,^5^ and the unmistakable effect this familiar toy has in eliciting the playful atmosphere of co‐creation that supports student engagement in the classroom.^6^, ^7^ Indeed, there is recent evidence that building dynamic physical models leads to better learning of key molecular biology concepts such as the Central Dogma when compared to other active learning approaches.^8^

The Lego Serious Play (LSP) methodology was introduced over 20 years ago by Roos and Victor as a business development tool used for strategy development, and later, team building.^9^ It is based on a timed, facilitated build‐share‐reflect cycle of building activities, called challenges that are undertaken according to a strict set of rules which now reside in the public domain.^10^ These start with skills‐building challenges for example,, ‘build a tower’ and build a duck’ to familiarize participants with the process and the rules, before build challenges allied to a particular goal are undertaken on an individual, and sometimes group, basis. These progressively more demanding initial skills‐building challenges are purposefully generic and simple so that participants can practice the LSP build‐share‐reflect cycle, use the rules and get used to building metaphors without worrying about the content or purpose of what they are building. This appears to be particularly important for scientists who may struggle with the abstract nature of the method because participants build *representations* or *metaphors* out of Lego bricks that are then related to the challenge (e.g., ‘build metabolism’) through the telling of the story of their object in the ‘share’ part of the cycle, and participants ask each other clarifying questions during the ‘reflect’ part of the cycle (Tables 1 and 2).

**TABLE 1:** Lego serious play rules and their purpose

Rule	Purpose	Alignment to Kahu's educational Interface for student engagement
Build: each session is timed by facilitator	Sense of purpose and progression through tasks, this is serious play	Emotional engagement: interest; Behavioral engagement: time and effort
Build: each challenge is set by facilitator	Retain the purpose of the session	Emotional engagement: interest; Behavioral engagement: time and effort
Build: trust your hands and the Lego	Supports the building of metaphorical objects later in the challenge by reducing over‐thinking	Cognitive engagement: deep learning and self‐regulation
Build/ Share: everyone builds, and everyone talks	Creates a sense of trust to support good quality sharing and reflection	Behavioral engagement: participation and interaction
Share: introduce and describe the object ‘your story’	The story relates the object built to the challenge and gives other participants something to reflect on	Emotional engagement: interest; Cognitive engagement: deep learning and self‐regulation; Behavioral engagement: participation and interaction
Share: everyone's contribution is equally important	Creates a sense of trust to support good quality sharing and reflection	Cognitive engagement: self‐regulation, behavioral engagement: participation and sense of belonging
Share: there is no right or wrong, only different perspectives	Creates a sense of trust to support good quality sharing and reflection	Cognitive engagement and sense of belonging
Reflect: Ask questions about the model not the person	Structured way into reflection for the participant, builds trust. Facilitator supports by asking clarifying questions and helping the dialogue serve the purpose of the session	Cognitive engagement: deep learning and self‐regulation. and sense of belonging

**TABLE 2:** Lego serious play build challenges

Build challenge	Purpose
1. Build a tower	Participants get used to timed building challenges with a simple task, sharing with others and reflecting on what they have built following the rules (skills building)
2. Build a duck from the blocks used to build a tower	More skills building practice, participants may need to start to practice the use of metaphor in their reflection if the blocks available lack resemblance to a duck (skills building)
3. Build a Good/ Bad Teacher using the same blocks	More skills building practice, participants practice the use of metaphor in their reflection (skills building). This task also brings in more elements of reflection and purpose into the build, in preparation for the main exercise.
4. Use any blocks you choose to build the life process you have been given individually: Metabolism: chemical processes that maintain life; Reproduction: production of new cells (cell division); Adaptation to the environment: gathers information and responds (cell signaling, gene expression); Homeostasis: maintains a constant internal environment (cytoskeleton & transport); Life also involves Growth: an increase in size or maturation	Use of metaphor required given abstract nature of build. Metaphor is helpful in this context as it allows participants to share and explore their understanding of these processes
5. Work together as a group. Use any blocks you choose to join together your individual processes into a whole cell	Group nature of build allows participants to negotiate their understanding of the interconnectivity of processes with their peers, learning from them. The facilitator's asking of clarifying questions allowed for understanding to be updated and missing links to be filled (by participants)

More recently, LSP began to be used in higher education (HE) settings as a means to explore and develop team building,^11^ identity^12^ and reflection.^13^ The method has also been modified and recognized as a form of object facilitation, used to support reflection on, and ultimately better understanding of, difficult or ‘threshold’ concepts in HE.^14^ Through the use of metaphor, object facilitation such as LSP facilitates successful application of strategies and mental models after knowledge acquisition and internalization has taken place, strengthening learning.^14^, ^15^ Therefore, LSP is ideally placed as a tool to support consolidation of learning for example, in revision tutorials.

Aspects of LSP such as the rules and use of metaphor/mental models may be mapped to aspects of student engagement using Kahu's integrated model^16^, ^17^ (Table 1). This model describes how various structural and psychosocial influences affect student engagement, such as university curriculum (e.g., what build challenges are set), student background, teaching (i.e., facilitation) and student skills and identity. They explain how students engage with their learning at educational institutions through emotions (such as interest and enthusiasm), cognitively (via deep learning and self‐regulation) and behaviorally (through participation, time and effort, and interaction). This results in immediate and longer‐term academic and social outcomes such as satisfaction, learning and community. This study explores the notion that LSP could be a useful method to facilitate student engagement with a formative revision exercise and therefore promote student understanding of complex biological systems, such as cells.

Over the past few years, the author has adapted LSP for use in a typical HE classroom setting: a first year Cell Biology course in a Scottish University, part of a four‐year undergraduate biological sciences honors degree. This context presents constraints, because less time, larger group sizes and a desire to improve accessibility prevent the use of the standard LSP technique.^10^ Here, a pilot project consisting of preliminary student and lecturer evaluations of an LSP Cell Biology revision exercise is presented, as a means of sharing this adapted method, and exploring its impact on student engagement in the hope of encouraging others to introduce this powerful tool to their practice.

## 2 METHOD

### 2.1 Ethics statement

All data were collected with University Research Integrity Committee approval [RIC0036].

### 2.2 Adaptations to traditional LSP method

Modifications from the standard LSP method are required to make the approach feasible in a classroom setting. For example, 3–6 groups were facilitated simultaneously as is usual for HE tutorials in order to manage large student numbers and listening circle approach was adopted to support this.^17^ The author acted as facilitator for all LSP tutorials at each table in a rotating manner, having used publicly available training documents to familiarize themselves with the background and theory before the session was designed.^10^ On some occasions, other lecturing staff on the module joined a table as a participant and aided with facilitation at that table, by enforcing the rules and asking clarifying questions. A group of peer practitioners play tested the modified method at a conference and fed back using a survey that was later modified to evaluate the pilot. Their feedback was incorporated before a small group of students play tested the updated method (and gave verbal feedback) before the modified method was implemented in the large class pilot presented below (Table 3).

**TABLE 3:** Modifications from standard LSP used in pilot study after student and peer feedback

Modification	Reason	Student/ peer feedback	Impact
Shorter session length (from 1 day to 2 h)	To facilitate timetabling and workload management	All builds could be accomplished in this time. Worked well in conjunction with reduced build time	Sessions were manageable within timetable and supported student engagement
Reduced build time for build tasks for example, Build a Tower/Duck from 5 min to 2	Contribute to a sense of momentum and focus	Peer conference playtest feedback was that original build times were too long. Reduced build times successful in student playtest and actual pilot	Constrained the time taken for the tutorial to an acceptable length for timetabled sessions. Successfully gave sense of momentum, reduced boredom and maintained engagement with task
Larger group sizes	To facilitate timetabling and workload management	Peer conference playtest feedback was that worked well in conjunction with introduction of listening circles	Sessions were manageable within timetable and supported student engagement
Introduction of listening circles: only one person in circle speaks at any one time to tell the story of their model, when speaker stops, others may ask clarifying questions, once speaker has used up their allotted time, they will ask the person to the left to tell the story of their model	To facilitate rules in larger groups (multiple tables) in the absence of the facilitator	Peer conference playtest feedback was that the approach worked well for reinforcing rules. Suggested use of buzzers so participants could reinforce rules, but student play test ruled these out as too intrusive	Fostered sense of trust and control amongst participants. Reinforced democratic ethos
The facilitator role evolved from enforcement of the ‘there is no right or wrong, only different perspectives’ rule to a more teaching‐focused role	To allow for questioning, coaching and correction of scientific inconsistencies and inaccuracies where necessary	Peer conference playtest feedback was that the original facilitation approach required modification if the teaching aims were to be met. The teaching‐focused role did not inhibit active participation in the student playtest	Potential conflict between democratic ‘no right or wrong’ ethos of LSP and requirements of the classroom. Mitigated by joining groups in co‐production of some tasks and maintaining focus of discussion on object, not person.
The role of facilitator moved away from ‘hands‐off’ LSP facilitation to include co‐production of some tasks with participants	To support an atmosphere of equality and inclusion in the classroom	This approach was tested in the student play‐test where students remarked positively on the facilitator's participation in the tasks. This did not inhibit active participation in the student play‐test	Helped to mitigate against negative aspects of a power‐dynamic that resulted from ‘hands‐off’ facilitation in the pilots
Inclusion of lecturing staff as participants in some groups	To train peers in facilitation	This approach was an extension to the co‐production facilitation trialed in the student play test (above). This did not seem to inhibit active participation in the pilot	Helped to reinforce facilitation over multiple groups. Supported an atmosphere of equality and inclusion in the classroom, although there is a potential conflict with the open and democratic ethos

### 2.3 Aims and learning outcomes

The aim of the formative LSP exercise was to provide an additional, engaging, opportunity for consolidation at the end of the course as a voluntary revision exercise. This provided students with an opportunity for formative feedback to support their learning on the Cell Biology course in preparation for the summative exam. Four out of five of the learning outcomes for the *course* were consolidated in the session:

1. LO1: Describe the structure and function of prokaryotic and eukaryotic cells and their component parts.
2. LO2: Outline the role of key processes within the cell, including cell division, and the role of metabolic pathways in the normal functioning of the cell.
3. LO3: Describe mechanisms by which information is processed, transferred and utilized to control cellular processes with special reference to cell signaling and transport.
4. LO4: Describe specialized adaptations of cells, both prokaryotic and eukaryotic, to their environments.

### 2.4 Participants

Participants were informed of the voluntary nature of the project and their right to withdraw at any time via the use of participant information sheets and informed consent. Students who did not wish to participate in the evaluation were free to take part in the exercise with no penalty. Participants were selected on a voluntary basis from the attendees of three, two‐hour long end of term formative (unassessed) LSP revision tutorials. In total 26 students took part in the exercise at the end of the spring trimester of 2017 together with their class lecturer. The 21 participants undertook the evaluation.

Participants were asked to revise at least one of the key concepts of metabolism, cell division, cell signaling, gene expression, cytoskeleton and transport before the session and were given no further prior knowledge of the exercise.

Students were divided into three tutorial groups of 40 students each for timetabling purposes. Student engagement with the tutorials was ~20%, in common with other formative end of term revision sessions within the programme. Participants were asked to sit in three self‐selected groups of 2–7. Each group had their own large table and LSP Lego set.

### 2.5 LSP‐based exercise

Once students were settled at their tables, the facilitator introduced themselves and the purpose of the session, taking care to explain that they were not a trained LSP facilitator and that the LSP method had been modified. Each table received a printed ‘about this session’ summary sheet (supplementary materials). Next, the rules and role of the facilitator, based on the modified LSP method^10^ were explained to the participants, together with the listening circle modification, again accompanied by a summary sheet for each table (Table 3, supplementary material).

The exercise began with timed ‘build a tower’ and ‘build a duck’ classic LSP skills building challenges that students undertook individually, sharing and reflecting within their groups^10^ (Tables 1 and 2). The next skills building challenge asked students to build a ‘good or bad teacher’ from the blocks used to create their duck. This transitional challenge introduced students to the idea of building metaphors for more complex ideas, placing the importance on the thinking and building process as well as the shared narrative once the artifact had been built.

Key concepts in Cell Biology were identified by the author after reflecting on over a decade of teaching first year undergraduates, some of whom are new to cell biology, and confirmed with two teaching colleagues on the course. These formed the basis of the next round of individual build challenges for the students. Linked concepts were combined for small groups and disaggregated for larger ones. They were printed on cards and one set was distributed per table. Individual students were asked to select the concept (below, in **bold**) they felt most confident with and spend 10 min building:

- **Metabolism**: chemical processes that maintain life
- Reproduction: production of new cells (**cell division**)
- Adaptation to the environment: gathers information and responds (**cell signaling, gene expression**)
- Homeostasis: maintains a constant internal environment (**cytoskeleton and transport**)
- **Growth**: an increase in size or maturation

The final build cycle consisted of students working together on each table to link together their concepts into a ‘cell’ (Table 2). During the final two build cycles, the facilitator was available to answer any questions students had about the concepts or the links between them. During the share part of the cycle, students explained the story of their build and how it related to the build challenge. The facilitator asked clarifying questions to coach the student groups into correcting any factual inaccuracies, a modification of the standard ‘there is no right or wrong, only different perspectives’ rule of LSP (Table 3).

### 2.6 Study aim

The aim of the study was to determine whether the modified LSP method supported student engagement with a revision tutorial. The study did not ask whether the tutorial resulted in any academic or social outcomes of engagement (such as improved grades), as this was out of scope.

### 2.7 Evaluation

A research assistant observed the LSP tutorials from the opposite side of the room and kept brief field notes. At the end of the tutorial the author/ facilitator distributed an anonymous paper survey to participants once the session had concluded Survey questions about the participants (questions 1–4) and expectation/ understanding of tasks (questions 5–7) were mapped to structural and psychosocial influences on student engagement, with questions about the experience of the task itself mapped to the states of engagement (questions 8–13, in order to understand the impact of the tutorial on student engagement.^16^ A mixture of closed, open and 7‐point Likert scale questions were used (Tables 4 and 5).

**TABLE 4:** Summary of survey questions and results relating to influences on engagement (participants)

Question number	Question	Mapping to Kahu's student engagement framework	Pilot study 100% completion rate for most questions except Qu 5 (Tower)
1	Are you an undergraduate student? Y/N If not, please state your role:	Structural Influences: background, Psychosocial Influences: Identity and skills	100% Yes
2	What is your age?	Structural Influences: background, Psychosocial Influences: Identity and skills	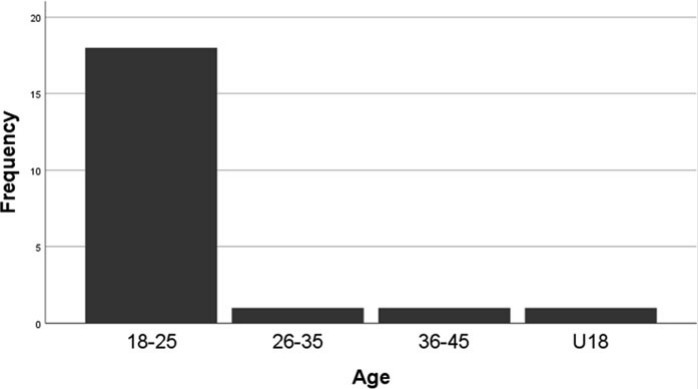
3	Before the task, how recently had you played with Lego?	Psychosocial Influences: Identity and Skills	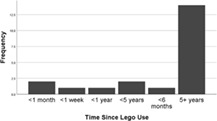
4	Before the task, had you ever played with Lego for a ‘serious’ purpose for example, workplace Y/N	Psychosocial Influences: Identity and Skills	28.6% Yes, 71.4% No

**TABLE 5:** Task evaluation: Task understanding

Question number	Question	Mapping to Kahu's student engagement framework	Pilot study	
5	I understood what I was expected to do for the task. Likert: 1–strongly disagree, 7–strongly agree	Psychosocial Influences: teaching, Skills	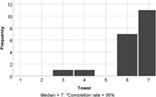	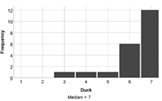
			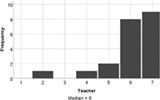	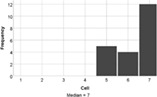
6	Do you have any other feedback you would like to share about the task?	Structural or Psychosocial Influences on Engagement	Open text, see Table 7	
7	The reality of the Lego tasks matched my expectation. Likert: 1–strongly disagree, 7–strongly agree	Psychosocial Influences: motivation; self‐efficacy	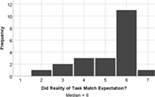	

In addition, a face‐to‐face semi‐structured interview with a participating member of lecturing staff on the course was conducted by the research assistant to identify perceived benefits and barriers to student experience of learning during and after the task. Initial analysis of the survey and interview were undertaken by the research assistant, with further qualitative analysis by the author.

All participant survey verbatim comments and lecturer interview data were analyzed using qualitative thematic analysis. A conventional, directed approach was applied, with coding based on Kahu's Student Engagement Framework.^16^ Themes were positive or negative engagement in any of the three domains (cognitive, affective and behavioral), and sub‐themes were determined by the implementation of the coding process. Each participant's response to each of the questions was individually coded, with the possibility that each response could be coded across multiple codes. The code book is shown in Tables 7 and 8.

## 3 RESULTS AND CONCLUSIONS

### 3.1 Participant characteristics–potential influences on engagement

80.7% participants completed the survey. Survey results show that 90.5% participating students were aged 18–25, with one student being under 18 and one in the 36–45 age range. Data collected from these two participants was in no way different that collected from the majority of the students and their data was included in the analysis.

Although some participants had more recent experience of using Lego, for 2/3rds students it had been over 5 years since they had experienced it. 28.6% participants had some experience of Lego use for serious purposes; these were distributed across the different categories of recency (Table 4).

### 3.2 LSP task evaluation–potential influences on engagement

On the whole, the students understood all of the build challenges well. One student recorded a score of 3/7 for the Understanding Tower question, with a different student recording the same score for the duck challenge. Each build challenge received a median understanding score of 6 or 7 indicating that participants understood the tasks well. Students agreed that the reality of the Lego tasks also matched expectations (median score = 6). Therefore, a lack of understanding is not expected to negatively influence student experience of engagement with the task.

### 3.3 LSP task evaluation–experiences of engagement

The experience of engagement with the LSP revision tutorial was very positively evaluated by the students, indeed all would recommend the tutorial to others (median score = 6), and two students requested more, similar tutorials in their feedback comments. The tutorial proved to be most effective at encouraging students to think creatively about biology (median score = 7), with positive impacts on all aspects of student engagement (interaction and understanding, Table 6).

**TABLE 6:** Task evaluation: Experience of task

Question number	Question	Mapping to Kahu's student engagement framework	Pilot study
8	This session improved my interaction with other students. Likert: 1–strongly disagree, 7–strongly agree	Behavioral Engagement: Interaction	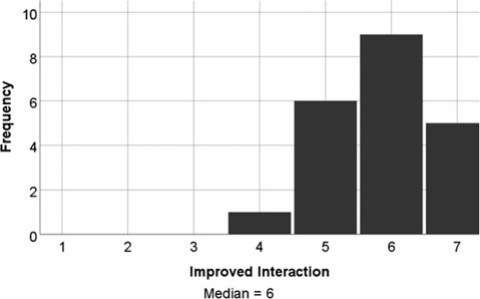
9	The session encouraged me to be creative in thinking about biology. Likert: 1–strongly disagree, 7–strongly agree	Cognitive Engagement: Deep Learning	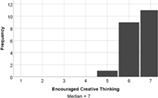
10	This session improved my understanding about cell biology. Likert: 1–strongly disagree, 7–strongly agree	Cognitive Engagement: Deep Learning, Proximal	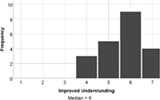
11	I would recommend this tutorial to others. Likert: 1–strongly disagree, 7–strongly agree	Emotional Engagement: Interest, Enthusiasm	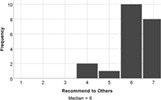
12	Do you have any other feedback you would like to share about how the impact of the Serious Play Lego tutorial on you?	States or outcomes of engagement	Open text, see Table 7
13	Please use the space below to share any other comments or feedback, thank you!	Structural or Psychosocial Influences on Engagement, States or outcomes of engagement	Open text, see Table 7

These findings were supported by student survey verbatim comments, which echo the positive experiences of creativity (*n* = 4), understanding (*n* = 5) and interaction/collaboration (*n* = 4) reported in the closed survey questions (Table 6). In addition, the use of metaphor/visualization emerged as the most frequent sub‐theme of cognitive engagement (*n* = 6). Comments were largely positive, and most were coded to the cognitive (*n* = 18) and affective (*n* = 16) domains of engagement, with five coded to the behavioral domain, and mostly related to interaction/ collaboration (Table 7). Students' experience of the task was reported as enjoyable (*n* = 6), interesting (*n* = 5) and different (*n* = 5), showing that students were also experiencing affective engagement. Negative comments were few (11% total comments) and none were aggregated into subthemes, indicating that negative experiences were not widespread, nor attributable to a specific cause (Table 7). However, two negative comments relate to exclusion from the task and difficulty with metaphors experienced by a student with additional learning needs. This indicates that the method requires further modification in order to become inclusive for all learners.

**TABLE 7:** Thematic analysis of free text survey responses in the cognitive, affective and behavioral domains of engagement

Theme	Positive or negative evaluation?	Sub‐theme	Number of discrete occurrences	Example quote
Cognitive	Positive	Metaphor/visualization	6	105: ‘It was really helpful to visualize parts of a cell and the process that occur within them’
		Understanding	5	201: ‘Tutorial helped me gain a better understanding of the link between each of the processes taught in the lecture’
		Creative/ thinking in different ways	4	
		Helped remember	3	102: ‘Helped relate course materials to metaphors and funny creations which helps me remember course content in exam situations’
	Negative	Metaphor/ visualization	1	107: ‘Neurodiversity should be considered ‐ Autistic people struggle with metaphors’
		Understanding/ purpose	1	
		Want more difficulty	1	
Affective	Positive	Fun/ enjoyable	6	205: ‘I really enjoyed it’
		Interesting/ involving	5	203: ‘I thought it was a good way to help learning as textbooks and lecture slides can become dull’
		Different	5	202: ‘It was interesting, different. Promoted our thinking.’ 103: ‘Refreshing to do something a little different in a difficult time like this [exam period]’
Behavioral/affective	Positive	Collaboration/ Group	4	104: ‘It was fun and helped to revise and come with ideas, working in a group.’
Behavioral/affective,	Negative	Collaboration/ Group	1	107: ‘As an autistic student I felt a bit excluded’
Behavioral	Positive	Good summarizing activity	1	110: ‘I think it was a good way to summarize and visualize all the topics learnt throughout the trimester’
Behavioral,	Negative	Want more discussion	1	
Feedback, positive		More please	2	
Feedback, negative		More Lego pieces needed	1	

### 3.4 Observation and lecturer interview themes

Interestingly, although most of the experiences related by students as verbatim comments were coded to the cognitive and affective experiences of engagement, the lecturer's interview responses focused mostly on positive *behavioral* aspects of engagement such as interaction and independence/empowerment in relation to the social and cognitive experience of learning (Table 8). This prevents triangulation of the student and lecturer responses, however this result is not surprising given the differing perspectives of the respondents. Taken together, this shows that LSP supports student engagement across all three domains of engagement.

**TABLE 8:** Thematic analysis of lecturer interview responses in the cognitive, affective and behavioral domains of engagement

Theme	Positive or negative evaluation?	Sub‐theme	Example quote
Cognitive	Positive	Creativity	‘And actually, I think that helps if you can… put a bit more fun into to, to allow that to happen more easily, because when they are thinking in a more fun, creative way, I think that it is when those connections happen more spontaneously.’ ‘Because I am a scientist, but that does not mean that… that I think in a really sort of like… I do not think in straight lines. I do not think in boxes, necessarily.’
Affective			
Behavioral	Positive	Social Interaction	
		Independent Learning	‘In the end we cannot keep… spoon feeding them the information.’ ‘There is a lot less input from, from the lecturer and from the person who's the facilitator. Which I think is great actually because they end up realizing that, that… they all know an awful lot more of what they think they know’.
		Empowerment	‘And this is what is so empowering about it, I think the fact that they are sharing a lot of information and they collectively know a lot of the answers already… and they just did not know that they knew the answers.’
	Negative	Play	‘Because, you know, if people think that, that they are sending their child to university and their child is playing […] they may not necessarily be happy their money is being spent on that. […] I suppose it's perception from the students, perception from the staff, perception [that] they are just playing, they are not doing anything.’

Themes of creativity and play were highlighted as particularly important aspects of to support student engagement: ‘I mean, I am teaching first year biology and I am trying to think of ways to engage them in cell biology, but actually quite a lot of what I have to do is to just literally tell them the basics.’ However, these were also identified as potential barriers to adoption of the LSP method because of perceived mismatches between playfulness and creativity and notions of serious scientific identity: ‘I think that perception of a scientist is somebody that you know, goes straight and thinks about things in a particular way. I think that a lot of what science is… is just making creative leaps and think creatively about solutions for things.’ There is acknowledgement that perception is flawed: ‘[…] There's lots of different flavors of science and scientist… and there's a lot of different flavors of creativity…’ whilst also identifying that there is likely to be a poor perception of the use of games and play in academic settings: ‘But, there will be people that will think, you know, it is all about airy‐fairy and kind of not particularly… well researched etcetera’.

A further logistical barrier to adoption of the LSP method was identified by the lecturer: ‘And when you are teaching first year classes it is really difficult to play games with them when you have got a huge, big class in front of you and you have got like two hours, you've got to get a certain level of understanding across.’ ‘We are all under pressure to teach, and we are all under pressure to teach certain things under, in a certain amount of time and when there is that sort of pressure and you have to do workshops on this or teach this and also help them prepare for assessments and a lot of us are just trying to find time to do this.’

### 3.5 Adaptations made to the LSP method

As a result of student and peer feedback from pilot sessions, the amount of time for each build task, the use of buzzers and the role of facilitator were modified before the method was implemented for the main data collection (Table 3).

The facilitator role necessarily evolved from the standard LSP enforcement of the ‘there is no right or wrong, only different perspectives’ rule, to a more teaching‐focused/coaching role which allowed for questioning, coaching and correction of scientific inconsistencies and inaccuracies where necessary. Care was taken to focus feedback correction empathetically on the scientific content of the student's narrative, and not on the student themselves to maintain the object orientation and democratic ethos of the approach.^18^ The role of facilitator was also modified after the pilot sessions to move away from traditional ‘hands‐off’ LSP facilitation to include co‐production of some tasks with participants. This helped to support an atmosphere of equality and inclusion in the classroom and helped to mitigate against negative aspects of a power‐dynamic that resulted from ‘hands‐off’ facilitation in the pilots.^18^

## 4 DISCUSSION

### 4.1 LSP as a method for supporting student engagement

The LSP‐like method appeared to support all aspects of student engagement, with students reporting most positive impact on cognitive and affective engagement measures such as understanding, interest and enjoyment (Tables 6 and 7), and the lecturer commenting most on behavioral engagement (Table 8). This supports the notion that the LSP‐like method fosters student understanding, that is, cognitive engagement, through the metaphor and visualization involved in this creative method for this group of students at least.^14^ However, it should be noted that the final build did incorporate some literal representations such as ladders for intracellular transport, which implies that there is a limitation to the usefulness of metaphor for students using the technique. The lecturer interview responses focused more on behavioral engagement (what the students did). This is not surprising given the external observer role that the lecturer took, and the focus of much of the gamification literature (of which LSP is arguably a part) on behavioral outcomes (e.g.,^19^).

### 4.2 Implementation and perspectives

Alterations made to the standard LSP method for application in the classroom setting do not seem to have had a negative impact on student experience of engagement in this study. Therefore, reducing the time given for build tasks and adopting a listing circle approach to facilitation, together with an active facilitator engaged in tasks as a peer may continue to be implemented. Indeed, this approach may augment the democratic ethos already present in the LPS method.^18^ However, one student comment highlighted that much more needs to be done to overcome neurological (and physical) barriers to engagement to improve accessibility of the method. An example I now use in my own practice is to make available on a voluntary basis a description of what the method entails in advance to the class in case anyone would like to plan. Lego now also manufacture Braille building blocks, and it would be possible to use other object facilitation materials (e.g., sand tray with objects as for sand play^20^) if dexterity is an issue.

One further observation is the usefulness of the ‘bad lecturer’ build challenge for gaining student feedback. This simple task allowed students to voice feedback in a different way to the usual survey and provided a rich discussion about student expectations and experiences of their first year of university. Again, the equality of voice supported by the method is likely to have played a role here, as seen in other education settings.^18^

### 4.3 Limitations and future work

This study concerns a small number of students on a first‐year undergraduate cell biology course in Scotland. The age range and level of skill and experience in the class is not necessarily representative of the student population as a whole, and a major limitation of the study is the double self‐selection of students, first by attending the tutorial and second by completing the survey. Because these structural and psychosocial influences on student engagement vary, caution is advised when generalizing any of the findings.^16^ Further studies are required to ascertain whether the benefits of the technique extend to other student contexts, cohorts or topics. In addition, before acting on potential barriers to implementation surfaced in the study, educator opinion must be more widely sought.

There are also limitations on the design of the study such that field notes about the skills‐building tasks were not kept, and the conversations between students carrying out their builds were not recorded. However, anecdotally, they revealed some unexpectedly rich information, and so it is recommended that field notes and recordings be made during any future study. Indeed, further exploration of the rich creative and social processes observed during the task would be helpful to confirm the themes of interaction, independence and empowerment revealed by the lecturer interview.

Although the changes made to the standard LSP method do not appear to affect student engagement in this task, it must be noted that the largest modification, to the role of facilitator, applied to one individual (the author), who facilitated all the sessions. Therefore, when applying a similar method elsewhere, facilitators are reminded that empathy and care must be taken to focus feedback correction on the scientific content of the student's *narrative*, and include elements of co‐production of some tasks with participants to maintain the object orientation and democratic ethos of the approach.^18^ Because of the importance of teaching and relationships as psychosocial influences on student engagement, the facilitation approach is likely to affect the student experience of engagement with the task.^16^ Therefore, if the positive benefits of LSP on student engagement are not recapitulated by a different facilitator, their approach should be re‐visited. Consider the perceived barriers to the adoption of LSP identified in the lecturer interview: a lack of evidence of efficacy of the technique together with issues around the identity of ‘serious’ scientists held by educators that potentially exclude notions of creativity and play have been recognized as issues for over a decade.^21^ Should the facilitator hold these views, this could affect their facilitation approach, and translate into a lack of student engagement in their teaching.

Interestingly, LSP has been used in education to explore professional identity,^19^ and future work may extend this work to examine the interaction between themes of creativity, play and scientific identity raised by this study. This is important given that creativity has recently been identified as playing a role in self‐efficacy and motivation in the STEAM (science, technology, engineering, arts and mathematics) classroom, and as an essential 21st century skill that must be present in our curricula.^22^

The evaluation in this study was carried out immediately after the activity and indicated that the method supports positive student engagement experiences. This warrants a follow up study to evaluate whether this experience of the task has a positive impact on long‐term student engagement outcome measures such as achievement of learning outcomes or satisfaction.^16^

Identifying whether the cell biology build tasks utilized in this study relate to any underlying threshold concepts in the course could open the door to the use of the Threshold Concepts framework to understand the processes at play during the exercise. Threshold concepts are those troublesome aspects of a discipline that, when crossed, are transformative for learning.^15^ They are integrative, irreversible, bounded and troublesome in nature, although not all troublesome concepts are threshold concepts.^14^ In Biology, language, scale, hypothesis building, variation, randomness, uncertainty and energy transformation have been put forward as threshold concepts, with energy transformation and scale being particularly relevant to this application.^23^, ^24^ In the more specialized area of cell and molecular biology, studies have focused on essential laboratory skills and graduate attributes, with limited attention paid to identifying threshold concepts. However, identifying threshold concepts has been discussed for biochemistry, and the Molecular Life Science Concept Inventory could also be powerful tool to explore relevant ideas with students.^25^, ^26^ Reframing this study in the context of threshold concepts could further transform the task, and better facilitate students crossing the threshold.^27^

## Supporting information


**Appendix** **S1**: Supporting Information

## References

[1] Templin MK , Mark A , Fetters . Meselson‐Stahl experimental simulation using Lego™ building blocks. Am Biol Teach. 2002;64(8):613–9. 10.2307/4451385

[2] Kirkpatrick G , Orvis K , Pittendrigh B . A teaching model for biotechnology and genomics education. J Biol Educ. 2002;37(1):31–5. 10.1080/00219266.2002.9655843

[3] Arís N , Orcos L . Educational robotics in the stage of secondary education: empirical study on motivation and STEM skills. Educ Sci. 2019;9(2):73. 10.3390/educsci9020073

[4] A working model of protein synthesis using Lego™ building blocks. Am Biol Teach. 2002;64(9):673–8. 10.2307/4451408

[5] Dorrell MI . Teaching Evo‐Devo with Legos: It's not the genes you have, It's how you use them. Am Biol Teach. 2019;81(2):120–5. 10.1525/abt.2019.81.2.120

[6] Zosh JM , Hopkins EJ , Jensen H , Liu C , Neale D , Hirsh‐Pasek K , Solis SL , Whitebread D . Learning through play: a review of the evidence (white paper). The LEGO Foundation, DK; 2017.

[7] Dann S . Facilitating co‐creation experience in the classroom with Lego serious play. Aus Market J (AMJ). 2018;26(2):121–31. 10.1016/J.AUSMJ.2018.05.013

[8] Newman DL , Stefkovich M , Clasen C , Franzen MA , Wright LK . Physical models can provide superior learning opportunities beyond the benefits of active engagements. Biochem Mol Biol Educ. 2018;46(5):435–44. 10.1002/bmb.21159 30281894 PMC6220871

[9] Roos J , Victor B . How it all began: the origins of LEGO® serious play®. Int J Manage Appl Res. 2018;5(4):326–43. 10.18646/2056.54.18-025

[10] D. Gauntlett , “Open‐source introduction to Lego Serious Play,” 2013. http://davidgauntlett.com/wp-content/uploads/2013/04/LEGO_SERIOUS_PLAY_OpenSource_14mb.pdf 20, 2019).

[11] Seidl T . Case study 1: playful team refection using LEGO® serious play®. Int J Game‐Based Learn. 2017;7(3):83–6. 10.4018/IJGBL.2017070108

[12] Tseng W‐C . An intervention using LEGO® SERIOUS PLAY® on fostering narrative identity among economically disadvantaged college students in Taiwan. J College Student Develop. 2017;58(2):264–82. 10.1353/csd.2017.0019

[13] Cavaliero T . ‘Creative blocs’: action research study on the implementation of Lego as a tool for reflective practice with social care practitioners. J Furth High Educ. 2017;41(2):133–42. 10.1080/0309877X.2015.1070396

[14] Barton G , James A . Threshold concepts, LEGO serious play® and whole systems thinking: towards a combined methodology. Pract Evidence Scholarship Teach Learn High Educ (PESTLHE). 2017;12:249–71. http://ualresearchonline.arts.ac.uk/8441/8/BartonandJAmes2017.pdf

[15] Rountree J , Robins A , Rountree N . Elaborating on threshold concepts. Comput Sci Educ. 2013;23(3):265–89. 10.1080/08993408.2013.834748

[16] Kahu ER . Framing student engagement in higher education. Stud High Educ. 2013;38(5):758–73. 10.1080/03075079.2011.598505

[17] Kahu ER , Picton C , Nelson K . Pathways to engagement: a longitudinal study of the first‐year student experience in the educational interface. High Educ. 2020;79(4):657–73. 10.1007/S10734-019-00429-W/FIGURES/1

[18] McCusker S . Everybody's monkey is important: LEGO® serious play® as a methodology for enabling equality of voice within diverse groups. Int J Res Method Educ. 2020;43:146–62. 10.1080/1743727X.2019.1621831

[19] Rivera ES , Garden CLP . Gamification for student engagement: a framework. J Furth High Educ. 2021;45:999–1012. 10.1080/0309877X.2021.1875201

[20] Amas D . We all love playing in the sand! Using sand play therapy in critical reflection with students in practice placement. J Pract Teach Learn. 2006;7(2):6–24. 10.1921/175951506784477690

[21] Barrow LH . Encouraging creativity with scientific inquiry. Creat Educ. 2010;1:1–6. 10.4236/ce.2010.11001

[22] Conradty C , Sotiriou SA , Bogner FX . How creativity in STEAM modules intervenes with self‐efficacy and motivation. Educ Sci. 2020;10(3):70. 10.3390/educsci10030070

[23] Batzli JM , Knight JK , Hartley LM , Maskiewicz AC , Desy EA . Crossing the threshold: bringing biological variation to the foreground. CBE—Life Sci Educ. 2016;15(4):es9. 10.1187/cbe.15-10-0221 27856553 PMC5132383

[24] Tibell LAE , Harms U . Biological principles and threshold concepts for understanding natural selection. Sci Educ. 2017;26(7–9):953–73. 10.1007/s11191-017-9935-x

[25] Howitt Susan WT , Trevor A , Manuel C , Susan H . A concept INVENTORY for molecular LIFE sciences: how WILL it help your teaching practice? Aus Biochem. 2008;39(3):14–7. https://core.ac.uk/download/pdf/55609916.pdf

[26] Loertscher J . Threshold concepts in biochemistry. Biochem Mol Biol Educ. 2011;39(1):56–7. 10.1002/bmb.20478 21433253

[27] Walck‐Shannon E , Batzli J , Pultorak J , Boehmer H . Biological variation as a threshold concept: can we measure threshold crossing? CBE—Life Sci Educ. 2019;18(3):ar36. 10.1187/cbe.18-12-0241 31418654 PMC6755314

