# Supplementary material for: Lego Serious Play: Building engagement with cell biology
Source: Biochem Mol Biol Educ. 2022 Jan 31;50(2):216–28. doi: 10.1002/bmb.21608 (PMC9303253; doi:10.1002/bmb.21608)
Supplement: Supplementary file 1 — Appendix S1: Supporting Information [file BMB-50-216-s001.docx]

Supplementary materials: Information sheets for LSP exercise given to students

1. **About this session**

- Facilitated by <Name>
- Part of <Course Name>
- Resulted with focus groups with students
- Aims to bring together core cell biology concepts and consolidate knowledge (revision)
- Optional participation in evaluation at end (research)
- Groups of 5/6

Caveats:

- I am not a trained facilitator
- This is not true Lego Serious Play

1. **The Rules**

- Facilitator sets challenge and time
- Build Share your Story Reflect and Learn
- Trust your hands and the Lego
- Everyone builds and everyone talks
- Everyone’s contribution is equally important
- Ask questions about the model, not the person
- There is no right or wrong, only different perspectives
- Please hand back your Lego at the end!

1. **The facilitator will**

- Set challenge and time
- Help the dialogue serve the purpose of the workshop
- Help you to reflect and share your story by asking clarifying questions
- Enforce the rules
- Act as a timekeeper

1. **The listening circle**

- Only one person in circle speaks at any one time to tell the story of their model
- When speaker stops, others may ask clarifying questions
- Once speaker has used up their allotted time, they will ask the person to the left to tell the story of their model

Remember the rules:

- Everyone’s contribution is equally important
- Ask questions about the model, not the person
- There is no right or wrong, only different perspectives

1. **Challenge 1: The Tower**

Detail: Use any Lego pieces you like to build your own, individual tower

Time: 5 minutes

1. **Challenge 2: The Duck**

Detail: Use any Lego pieces you like to build your own, individual duck

Time: 5 minutes

1. **Challenge 3: The Teacher**

Detail: Use the Lego you used to build your duck to build something that shows a good or bad teacher/ lecturer. You may use a few additional blocks if you wish.

Time: 5 minutes

1. **Challenge 4: Build a Cell**

Background: Cells are the basic unit of life. Life has been defined as the sum total of all of these processes:

- **Metabolism**: chemical processes that maintain life
- Reproduction: production of new cells (**cell division & DNA replication**)
- Adaptation to the environment: gathers information and responds (**cell signalling, gene expression**)
- Homeostasis: maintains a constant internal environment (**cytoskeleton & transport**)

Life also involves the following processes that you may choose to touch upon:

- Growth: an increase in size or maturation
- Mutation and repair

Please choose one of the processes **highlighted in bold** above. Each group should have one each of these.

Part 1

Detail: Use any blocks you choose to build the life process you have chosen individually. Ask the facilitator to explain anything you don’t understand. Remember let your hands do the thinking!

Time: 10 minutes

Part 2

Detail: Work with the rest of your table to link together your processes into one big cell.

Time: 10 minutes
